# Relationship between preoperative high intraocular pressure and retinal nerve fibre layer thinning after glaucoma surgery

**DOI:** 10.1038/s41598-019-50406-7

**Published:** 2019-09-25

**Authors:** Woo-Jin Kim, Kyoung Nam Kim, Jae Yun Sung, Jung Yeul Kim, Chang-sik Kim

**Affiliations:** 1 Department of Ophthalmology, Chungnam National University Hospital, Daejeon, Korea; 2 Department of Ophthalmology, Chungnam National University College of Medicine, Daejeon, Korea

**Keywords:** Optic nerve diseases, Risk factors

## Abstract

Recent reports show varying results regarding peripapillary retinal nerve fibre layer (RNFL) thickness after intraocular pressure (IOP)-lowering glaucoma surgery. We hypothesised that different levels of the preoperative IOP influence RNFL thickness. A total of 60 patients (60 eyes) with glaucoma, who underwent glaucoma surgery and had a stable postoperative mean IOP < 22 mmHg, were enrolled. The RNFL thickness was measured using spectral domain optical coherence tomography, before and at 3–6 months after surgery. The preoperative peak IOP, 37.4 ± 10.8 mmHg, decreased to a postoperative mean IOP of 14.8 ± 3.5 mmHg (p < 0.001). The average RNFL thickness was significantly reduced from 75.6 ± 17.7 μm to 70.2 ± 15.8 μm (p < 0.001). In subgroup analyses, only patients with a preoperative peak IOP ≥ median value (37 mmHg) exhibited significant RNFL thinning (9.7 ± 6.6 μm, p < 0.001) associated with a higher preoperative peak IOP (r = 0.475, p = 0.008). The RNFL thinning was evident for a few months after glaucoma surgery in patients with a higher preoperative peak IOP, although the postoperative IOP was stable.

## Introduction

Glaucoma, the most common chronically progressive optic neuropathy, is a leading cause of complete blindness^1^. High intraocular pressure (IOP) is a well-known risk factor for the onset and progression of glaucoma^2–4^. Furthermore, IOP lowering is the only effective treatment for glaucoma^5,6^. Glaucomatous damage is generally irreversible, so the goal of IOP lowering treatment is not improvement, but rather prevention or slowing, of disease progression. However, recent studies, not only in children but also in adults, have reported structural recovery, such as optic disc cupping reversal and reversal of lamina cribrosa deepening, following IOP-lowering treatment^7–10^. Reports of changes in retinal nerve fibre layer (RNFL) thickness after IOP-lowering treatment have been equivocal. Ely *et al*. reported that some paediatric glaucoma patients with optic disc cupping reversal resulting from decreased IOP after glaucoma surgery showed continued postoperative RNFL thinning^9^. Chang and Grajewski reported paradoxical RNFL thinning after cupping reversal following trabeculotomy^11^. By contrast, some studies have shown a significant increase in RNFL thickness following IOP-lowering surgery, or treatment with IOP-lowering agents^12–15^. However, some other studies have reported no RNFL thickness change upon lowering IOP^10,16,17^.

Some glaucoma patients in our clinic showed considerable thinning of the RNFL, as measured using spectral domain optical coherence tomography (SD-OCT), a few months after glaucoma surgery, although their postoperative IOP was well controlled. We were concerned that this RNFL thinning denoted worsening glaucoma, and wondered if such thinning reflected the actual progression of glaucoma. It is important to determine whether postoperative RNFL thinning, as detected by SD-OCT, reflects actual deterioration, because RNFL thickness measured using SD-OCT is considered a reliable and objective marker of glaucoma progression^18,19^.

The first objective of the present study was therefore to identify whether a significant reduction in RNFL thickness occurred during the early postoperative period and whether clinical factors such as preoperative IOP were associated with changes in RNFL thickness. The second objective was to determine whether changes in the macular ganglion cell plus inner plexiform layer (GCIPL) thickness and in the visual field occurred following surgery, which are also essential parameters in assessing glaucoma progression^20–22^.

## Results

Sixty eyes of 60 patients were enrolled in the study. The clinical characteristics of these patients are listed in Table 1. The mean age of the patients was 55.4 ± 13.6 years, and 45 patients were male. The average preoperative peak IOP was 37.4 ± 10.8 mmHg. Twenty-nine patients were diagnosed with POAG; the other patients were diagnosed with chronic angle-closure glaucoma, uveitis-associated glaucoma, pseudoexfoliation glaucoma, or steroid-induced glaucoma. The mean follow-up period was 12.4 ± 1.2 months.

**Table 1:** Clinical characteristics of the study patients.

Clinical characteristic	
Age (y)	55.4 ± 13.6 (18–75)
Sex (male/female)	45/15
Best-corrected visual acuity (logMAR)	0.1 ± 0.2 (−0.3–0.3)
Spherical equivalent (D)	−1.4 ± 1.9 (−8.5–2.0)
Axial length (mm)	23.6 ± 1.7 (21.6–27.4)
Central corneal thickness (µm)	538.2 ± 33.3 (486–606)
Disc area (mm^2^)	2.2 ± 0.4 (1.52–2.98)
Disc haemorrhage presence, n (%)	5 (8.3)
Diagnosis, n (%)	
Primary open-angle glaucoma	29 (48.3)
Chronic angle-closure glaucoma	9 (15.0)
Uveitis associated glaucoma	9 (15.0)
Pseudoexfoliation glaucoma	8 (13.3)
Steroid induced glaucoma	5 (8.3)
Preoperative peak IOP (mmHg)*	37.4 ± 10.8 (20–58)
Average RNFL thickness (µm)	75.6 ± 17.7 (54–112)
Average GCIPL thickness (µm)	66.7 ± 12.6 (45–97)
Mean deviation (dB)	−19.3 ± 9.6 (−31.0–−1.5)
Pattern standard deviation (dB)	6.5 ± 3.1 (1.72–15.3)
Visual field index (%)	54.3 ± 33.2 (2.0–98.0)
Follow-up period (months)	12.4 ± 1.2 (10.0–15.0)

logMAR = logarithm of the minimum angle of resolution, D = dioptres, IOP = intraocular pressure, RNFL = retinal nerve fibre layer, GCIPL = ganglion cell layer plus inner plexiform layer.

Continuous variables are expressed as means ± standard deviation (range).

^*^The highest IOP among those measured between 1 month and 1 day before surgery.

Table 2 shows changes in clinical parameters indicating the severity of the glaucoma following trabeculectomy or Ahmed valve implantation. The IOP significantly decreased from 37.4 mmHg to 14.8 mmHg (p < 0.001). The average RNFL thickness showed a significant reduction, from 75.6 µm to 70.2 µm (p < 0.001). There was no significant change in visual acuity, average GCIPL thickness, mean deviation, pattern standard deviation, or visual field index.

**Table 2:** Comparison of preoperative and postoperative clinical parameters indicating glaucoma severity.

	Preoperative value	Postoperative value	p-value
Best-corrected visual acuity (logMAR)	0.2 ± 0.2	0.1 ± 0.2	0.625
Intraocular pressure (mmHg)	37.4 ± 10.8*	14.8 ± 3.5†	<0.001
Average RNFL thickness (µm)	75.6 ± 17.7	70.2 ± 15.8	<0.001
Average GCIPL thickness (µm)	68.0 ± 13.7	66.9 ± 12.7	0.145
Mean deviation (dB)	−19.3 ± 9.6	−18.7 ± 10.4	0.800
Pattern standard deviation (dB)	6.5 ± 3.1	6.8 ± 3.3	0.354
Visual field index (%)	54.3 ± 33.2	54.6 ± 34.1	0.813

logMAR = logarithm of the minimum angle of resolution, RNFL = retinal nerve fibre layer, GCIPL = ganglion cell layer plus inner plexiform layer.

Continuous variables are expressed as means ± standard deviation.

^*^Preoperative peak intraocular pressure (IOP) was the highest IOP among those measured between 1 month and 1 day before surgery.

^†^Postoperative mean IOP was calculated as the sum of all IOPs recorded during the postoperative period commencing 1 month postoperatively divided by the total number of examinations, up until the postoperative ophthalmic examination.

Table 3 lists changes in the peripapillary RNFL thickness and macular GCIPL thickness following glaucoma surgery. The change in average RNFL thickness was −5.4 µm (p < 0.001). By quadrant, superior, inferior, and temporal RNFL thicknesses significantly decreased, by 8.0 ± 13.5 µm, 8.0 ± 11.1 µm, and 5.4 ± 9.6 µm, respectively (all, p < 0.001). There was no significant change in average or sectoral GCIPL thickness parameters.

**Table 3:** Changes in peripapillary RNFL thickness and macular GCIPL thickness after glaucoma surgery (means ± SD, µm).

	Preoperative value	Postoperative value	Changes	p-value
Average RNFL thickness	75.6 ± 17.7	70.2 ± 15.8	−5.4 ± 6.9	**<0.001**
Quadrant RNFL thickness				
Superior	87.1 ± 25.1	79.1 ± 25.5	−8.0 ± 13.5	**<0.001**
Nasal	66.3 ± 10.6	64.3 ± 13.3	−2.0 ± 12.3	0.215
Inferior	84.9 ± 28.7	76.8 ± 24.1	−8.0 ± 11.1	**<0.001**
Temporal	63.9 ± 15.6	58.5 ± 14.4	−5.4 ± 9.6	**<0.001**
Average GCIPL thickness	68.0 ± 13.7	66.9 ± 12.7	−1.1 ± 5.8	0.145
Sectoral GCIPL thickness				
Superior	68.2 ± 17.2	67.8 ± 15.3	−0.4 ± 12.1	0.813
Superonasal	69.3 ± 17.7	68.2 ± 15.4	−1.1 ± 12.9	0.501
Inferonasal	68.0 ± 15.7	66.5 ± 13.8	−1.5 ± 8.6	0.199
Inferior	64.7 ± 14.1	64.4 ± 13.0	−0.3 ± 6.1	0.716
Inferotemporal	67.2 ± 17.8	66.3 ± 15.5	−0.9 ± 8.8	0.448
Superotemporal	68.4 ± 15.2	67.2 ± 14.8	−1.2 ± 9.1	0.332

SD = standard deviation, RNFL = retinal nerve fibre layer, GCIPL = ganglion cell layer plus inner plexiform layer.

Table 4 lists the clinical parameters associated with postoperative thinning of the average RNFL thickness. Univariate analyses identified better (less damage) preoperative clinical parameters associated with the severity of glaucoma, including the average RNFL thickness (p < 0.001), average GCIPL thickness (p = 0.022), mean deviation (p = 0.008), and visual field index (p = 0.015), which were all significantly associated with a greater postoperative reduction in average RNFL thickness. Higher preoperative peak IOP (p < 0.001) and IOP reduction (p < 0.001) were significantly associated with a greater reduction in average RNFL thickness. However, postoperative mean IOP was not associated with thinning of the RNFL after surgery (p = 0.451). In multivariate analyses, the average RNFL thickness decreased by 1.25 µm after surgery for every 10 µm of additional preoperative RNFL thickness (p = 0.038), and decreased by 3.38 µm after surgery for every additional 10 mmHg of preoperative peak IOP (p < 0.001). The variance inflation factor (VIF) was 1.231, indicating that there was no collinearity between two variables^23^.

**Table 4:** Clinical factors associated with postoperative thinning of the average retinal nerve fibre layer thickness (µm).

	Univariate			Multivariate		
	β	95% CI	p-value	β	95% CI	p-value
Age (per 1 y older)	−0.088	−0.215–0.039	0.181			
Sex (male)	−1.156	−5.186–2.874	0.576			
Axial length (per 1 mm longer)	0.139	−0.857–1.135	0.290			
Central corneal thickness (per 1 µm thicker)	0.007	−0.077–0.091	0.509			
Disc area (per 1 mm2 larger)	1.002	−2.979–4.983	0.363			
Disc haemorrhage (presence)	0.135	−1.186–1.456	0.601			
Preoperative severity of glaucoma						
Average RNFL thickness (per 1 µm thicker)	0.180	0.092–0.268	**<0.001**	0.125	0.011–0.239	**0.038**
Average GCIPL thickness (per 1 µm thicker)	0.147	0.025–0.269	**0.022**			
Mean deviation (per 1 dB higher)	0.283	0.083–0.483	**0.008**			
Visual field index (per 1% higher)	0.073	0.018–0.128	**0.015**			
IOP related parameters						
Preoperative peak IOP (per 1 mmHg higher)^*^	0.435	0.317–0.553	**<0.001**	0.338	0.175–0.501	**<0.001**
Postoperative mean IOP (per 1 mmHg higher)^†^	−0.192	−0.688–0.304	0.451			
IOP reduction (per 1 mmHg higher)^‡^	0.399	0.287–0.511	**<0.001**			
IOP fluctuation (per 1 mmHg higher)	−0.045	−0.352–0.262	0.836			

CI = confidence interval, IOP = intraocular pressure, RNFL = retinal nerve fibre layer, GCIPL = ganglion cell layer plus inner plexiform layer, IOP = intraocular pressure.

Average RNFL thickness and preoperative peak intraocular pressure (IOP) remained significantly different in multivariate regression analysis (stepwise selection method). The variance inflation factor was 1.231, such that there was no collinearity between any two variables.

^*^The highest IOP among those measured between 1 month and 1 day before surgery.

^†^The IOP calculated as the sum of all IOPs recorded during the postoperative period commencing 1 month postoperatively divided by the total number of examinations, up until the postoperative ophthalmic examination.

^‡^Preoperative peak IOP–postoperative mean IOP.

### Subgroup analyses according to preoperative peak IOP (≥37 mmHg vs. <37 mmHg)

**Figure 1:**
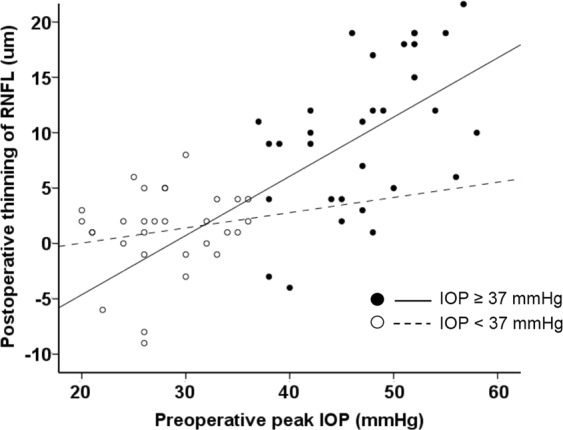
Correlation between the preoperative peak intraocular pressure (IOP) and postoperative thinning of the retinal nerve fibre layer (RNFL). In patients with IOP ≥ 37 mmHg (median value), postoperative RNFL thinning was significantly associated with preoperative peak IOP (r = 0.475, p = 0.008). In patients with IOP < 37 mmHg, there was not a significant correlation between postoperative RNFL thinning and preoperative peak IOP (r = 0.175, p = 0.354).

The postoperative reduction in the average RNFL thickness was significantly correlated with preoperative peak IOP (r = 0.689, p < 0.001). In subgroup analyses, patients were divided into two groups based on their median preoperative peak IOP: in 30 patients with an IOP ≥ 37 mmHg (group 1), postoperative RNFL thinning was significantly associated with preoperative peak IOP (r = 0.475, p = 0.008). In the other 30 patients with IOP < 37 mmHg (group 2), there was no significant correlation between postoperative RNFL thinning and preoperative peak IOP (r = 0.175, p = 0.354, Fig. 1).

Tables 5 and 6 compare demographics and changes following surgery between the two groups, respectively. Preoperative peak IOPs were 46.7 mmHg in group 1 and 28.0 mmHg in group 2 (p < 0.001). The postoperative mean IOP did not differ between the two groups (14.8 mmHg vs. 14.7 mmHg, respectively, p = 0.860). The number of POAG patients was significantly higher in group 2 than in group 1 (p = 0.002). The number of uveitis-associated glaucoma patients was significantly lower in group 2 than in group 1 (p = 0.026). The preoperative average RNFL thickness of group 1 was greater than that of group 2 (81.0 µm vs. 70.1 µm, p = 0.016). The preoperative average GCIPL thickness did not differ between the two groups (70.1 µm vs. 65.9 µm, respectively, p = 0.246), nor did the postoperative average RNFL thickness or GCIPL thickness. The changes in average RNFL thickness and average GCIPL thickness following surgery were significant only in group 1 (−9.7 µm, p < 0.001 and −2.4 µm, p = 0.030, respectively). The average extent of RNFL thinning differed significantly between groups 1 and 2 (p < 0.001), but that of GCIPL thinning did not (p = 0.113). The preoperative and postoperative mean deviation and visual field index did not differ between the two groups, and there was no significant change in the mean deviation or visual field index in either group.

**Table 5:** Comparison of demographic characteristics between patients with higher versus lower preoperative peak IOP

	Preoperative peak IOP ≥ 37 mmHg (n = 30)	Preoperative peak IOP < 37 mmHg (n = 30)	p- value
Age (y)	52.9 ± 15.2	58.0 ± 11.6	0.151
Sex (male/female)	23/7	22/8	1.000^*^
Best-corrected visual acuity (logMAR)	0.1 ± 0.2	0.2 ± 0.2	0.517
Spherical equivalent (D)	−1.4 ± 2.1	−1.3 ± 1.8	0.879
Axial length (mm)	23.5 ± 2.0	23.6 ± 1.1	0.627
Central corneal thickness (µm)	542.8 ± 32.7	533.6 ± 27.5	0.283
Disc area (mm^2^)	2.1 ± 0.5	2.2 ± 0.4	0.471
Disc haemorrhage presence, n (%)	1 (3.3)	4 (13.3)	0.353^*^
Diagnosis, n (%)			
Primary open-angle glaucoma	8 (26.7)	21 (70.0)	0.002^*^
Chronic angle-closure glaucoma	6 (20.0)	3 (10.0)	0.472^*^
Uveitis-associated glaucoma	8 (26.7)	1 (3.3)	0.026^*^
Pseudoexfoliation glaucoma	5 (16.7)	3 (10.0)	0.706^*^
Steroid-induced glaucoma	3 (10.0)	2 (6.7)	1.000^*^
Preoperative peak IOP (mmHg)	46.7 ± 5.9	28.0 ± 4.9	< 0.001

IOP = intraocular pressure, logMAR = logarithm of the minimum angle of resolution, D = dioptres.

^*^Fisher’s exact test.

**Table 6:** Comparison of clinical parameters between patients with higher versus lower preoperative peak IOP (mean ± SD, µm).

	Preoperative peak IOP ≥ 37 mmHg (n = 30)	Preoperative peak IOP < 37 mmHg (n = 30)	p-value
IOP parameters (mmHg)			
Preoperative peak IOP	46.7 ± 5.9	28.0 ± 4.9	<0.001
Postoperative mean IOP	14.8 ± 3.4	14.7 ± 3.8	0.860
IOP reduction	31.9 ± 6.8	13.3 ± 7.0	<0.001
p-value	<0.001	<0.001	
Average RNFL thickness (µm)			
Preoperative	81.0 ± 18.7	70.1 ± 15.0	0.016
Postoperative	71.4 ± 16.5	69.0 ± 15.2	0.560
Change	9.7 ± 6.6	1.1 ± 3.8	<0.001
p-value	<0.001	0.113	
Average GCIPL thickness (µm)			
Preoperative	70.1 ± 15.4	65.9 ± 12.7	0.246
Postoperative	67.6 ± 12.6	65.8 ± 12.8	0.591
Change	2.4 ± 5.8	0.2 ± 4.8	0.113
p-value	0.030	0.813	
Mean deviation (dB)			
Preoperative	−18.1 ± 9.9	−20.5 ± 10.6	0.303
Postoperative	−17.3 ± 8.9	−20.0 ± 11.5	0.338
Change	−0.8 ± 3.2	−0.5 ± 3.4	0.512
p-value	0.625	0.733	
Visual field index (%)			
Preoperative	55.4 ± 28.9	53.2 ± 32.7	0.437
Postoperative	55.3 ± 28.7	53.9 ± 36.1	0.301
Change	0.1 ± 9.2	−0.7 ± 10.0	0.101
p-value	0.907	0.495	

SD = standard deviation, IOP = intraocular pressure, RNFL = retinal nerve fibre layer, GCIPL = ganglion cell layer plus inner plexiform layer.

**Figure 2:**
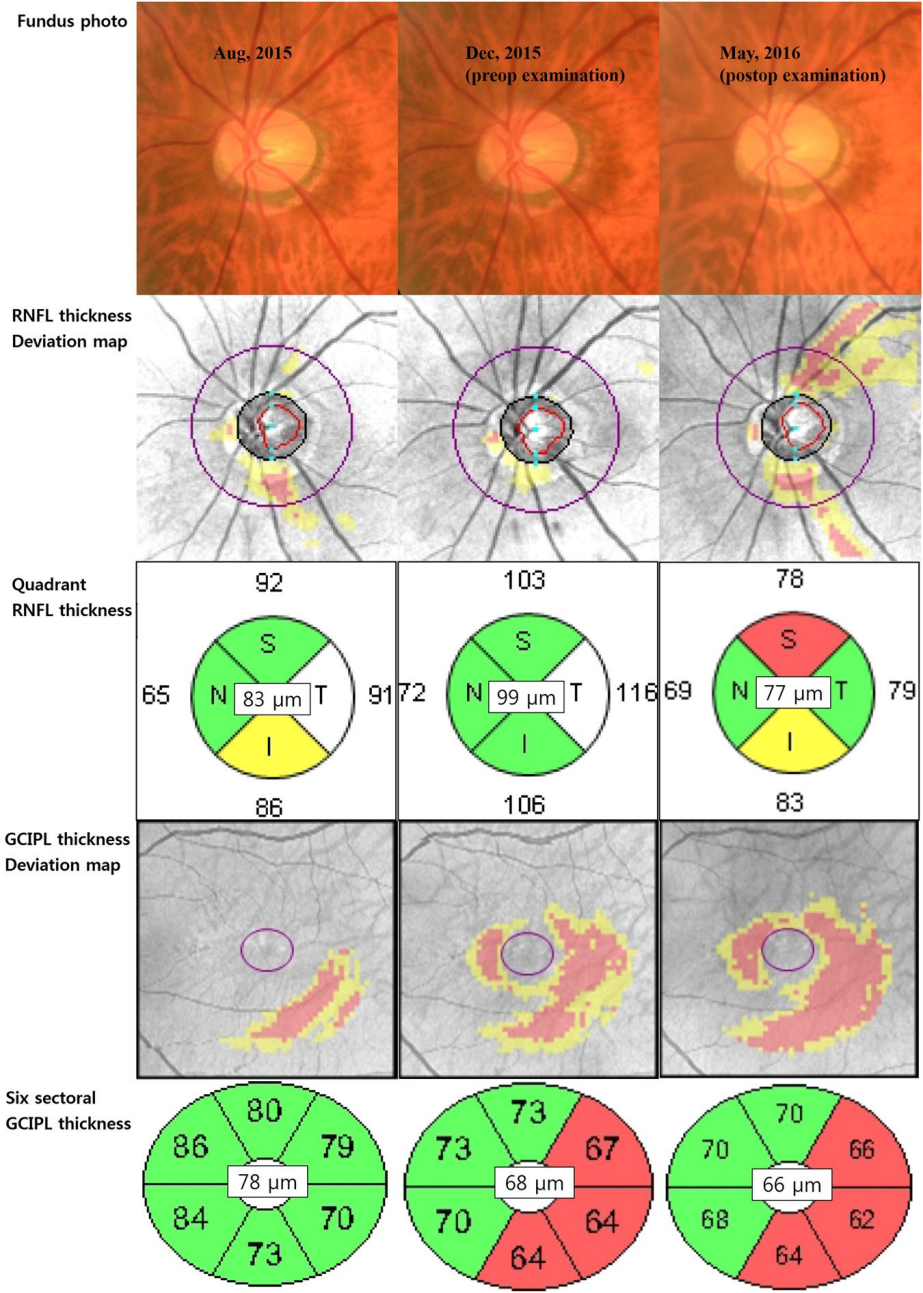
A representative patient. He was 72 years old and had primary open-angle glaucoma in both his eyes. Although the usual intraocular pressure in the left eye was uncontrolled, ranging 25~30 mmHg despite IOP lowering medical therapy, he wanted to delay surgical treatment. However, he eventually underwent trabeculectomy because of the more increased IOP in his left eye. His preoperative peak IOP was 57 mmHg and the average RNFL thickness was 99 µm (it was 16 µm thicker than the average RNFL thickness of 83 µm measured 4 months previously). Five months after the trabeculectomy, his average RNFL thickness decreased by 22 µm. During the same period, the preoperative average GCIPL thickness decreased by only 2 µm. RNFL = retinal nerve fibre layer, GCIPL = ganglion cell plus inner plexiform layer, T = temporal, S = superior, N = nasal, and I = inferior.

Figure 2 shows a representative patient. He was 72 years old and had POAG in both his eyes. Although his usual IOP in the left eye was uncontrolled, ranging 25–30 mmHg despite IOP lowering medical therapy, he wanted to delay surgical treatment. However, he eventually underwent trabeculectomy because of the more increased IOP in his left eye. His preoperative peak IOP was 57 mmHg and the average RNFL thickness was 99 µm (it was 16 µm thicker than the average RNFL thickness of 83 µm measured 4 months previously). Five months after the trabeculectomy, his average RNFL thickness decreased by 22 µm. During the same period, the preoperative average GCIPL thickness decreased by only 2 µm.

### Subgroup analyses according to glaucoma type: POAG vs. glaucoma other than POAG

**Figure 3:**
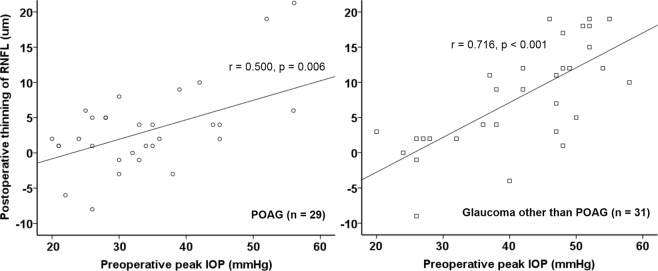
Correlation between preoperative peak IOP and postoperative thinning of the RNFL in patients with primary open-angle glaucoma (POAG, n = 29) and with glaucoma other than POAG (n = 31). In patients with POAG, postoperative RNFL thinning was significantly associated with the preoperative peak IOP (r = 0.500, p = 0.006). In patients with glaucoma other than POAG, postoperative RNFL thinning was also significantly associated with the preoperative peak IOP (r = 0.716, p < 0.001).

Patients were divided into two groups based on glaucoma type: there were 29 patients in the POAG group and 31 in the glaucoma-other-than-POAG group. The preoperative peak IOP differed between the two groups (33.0 ± 9.3 mmHg vs. 41.5 ± 10.7 mmHg, respectively, p = 0.003). The postoperative mean IOP did not differ between the two groups (14.1 mmHg ± 2.7 vs. 15.3 ± 4.1 mmHg, respectively, p = 0.113). The preoperative average RNFL thickness was greater in the ‘other glaucoma’ group (68.8 ± 16.9 µm vs. 81.9 ± 16.3 µm, p = 0.003). The preoperative average GCIPL thickness did not differ between the two groups (65.9 ± 13.8 µm vs. 70.0 ± 13.5 µm, respectively, p = 0.216). In the POAG group, there was a significant reduction in the average RNFL thickness 2.8 ± 5.1 µm (p = 0.005), but there was no significant reduction in the average GCIPL thickness 1.0 ± 5.4 µm (p = 0.353). These results were similar in the other glaucoma group; the RNFL thinned by 7.9 ± 7.4 µm (p < 0.001), and the GCIPL by 1.2 ± 6.2 µm (p = 0.138). Thinning of the average RNFL thickness was significantly associated with preoperative peak IOP in both the POAG group and the ‘other glaucoma’ group (r = 0.500, p = 0.006, and r = 0.716, p < 0.001, respectively, Fig. 3).

## Discussion

This study found no significant changes in GCIPL thickness or visual field parameters, with a significant reduction seen only in average RNFL thickness during the early postoperative period. The magnitude of RNFL thinning was positively associated with preoperative peak IOP, but not with postoperative mean IOP. Based on these results, we speculate that, at least in part, this postoperative RNFL thinning might reflect resolution of retinal nerve fibre swelling, rather than progression of glaucomatous optic neuropathy.

Some previous studies have reported results that conflict with our findings. Aydin *et al*.^14^ found significant postoperative RNFL thickening (median of 9.9 µm) using a prototype OCT. Yamada *et al*. also found a postoperative increase in RNFL thickness (mean of approximately 6 µm) using scanning laser polarimetry^13^. More recent studies have reported no meaningful change in RNFL thickness postoperatively^10,16,17^. We suggest that the possible causes of the equivocal results are as follows. First, in the previous studies, the instrument used for RNFL thickness measurement differed from that used in the present investigation^13–17^. Chang *et al*.^16^ and Rebolleda *et al*.^17^ used time-domain OCT, which is an older version of OCT with lower axial resolution than SD-OCT (10 µm vs. 5 µm)^24–26^. It is therefore possible that the amount of RNFL thinning measured in the present study (5.4 µm) was not detected in the previous study. Second, the preoperative IOPs of the enrolled patients differed^10,13–15,17^. In subgroup analyses based on preoperative peak IOPs, there was significant RNFL thinning, and the higher the preoperative peak IOP, the greater the postoperative RNFL thinning in patients with a preoperative peak IOP ≥ 37 mmHg (median value). However, there was no meaningful change in RNFL thickness and no correlation was found between preoperative peak IOP and the extent of RNFL thinning in patients with a preoperative peak IOP < 37 mmHg. The mean preoperative IOPs in the studies by Aydin *et al*.^14^ and Yamada *et al*.^13^ were 22.0 ± 6.4 mmHg (38 eyes) and 22.6 ± 6.9 mmHg (46 eyes), respectively, even lower values than in our subgroup with a mean preoperative peak IOP < 37 mmHg (28.0 ± 4.9 mmHg in 30 eyes). The mean preoperative IOPs reported by Sarkar *et al*.^15^ and Rebolleda *et al*.^17^ were 26.9 ± 6.4 mmHg and 23.6 ± 5.1 mmHg, respectively. In Waisbourd *et al*.^10^, only 8 of 62 eyes had an IOP higher than 32 mmHg. In addition, as in our study, they did not measure the postoperative axial length of the eye. When IOP decreases, the axial length decreases^27,28^. With a shorter axial length, the RNFL is thicker^29,30^. The postoperative increase in RNFL thickness found in the previous studies, and even among some patients in our study, may have been affected by the reduced axial length after surgery (Fig. 1). In the same context, the postoperative RNFL thinning observed in our study might have been greater than the measured values. Although the mean preoperative (31.5 ± 8.2 mmHg) and postoperative (12.8 ± 4.6 mmHg) IOPs in the study of Chang *et al*. were similar to those in our study, a high preoperative IOP (≥37 mmHg) was noted in only five patients (23.8%), whereas in our study, 30 patients (50%) had IOPs ≥ 37 mmHg. Although there was no significant change in postoperative RNFL thickness when considering all patients, one case in the study by Chang *et al*. showed postoperative RNFL thinning of more than 15 µm, and his preoperative IOP was approximately 50 mmHg^16^.

Some studies have reported RNFL thinning after IOP reduction, similar to our results. Ely *et al*. reported that some paediatric glaucoma patients with IOP reductions and cupping reversal after glaucoma surgery showed continued RNFL thinning^9^. In their study, the mean preoperative IOP in nine eyes with preoperative and postoperative OCT data was 34.2 ± 6.6 mmHg, and the RNFL thinned by 8.6 ± 18.6 µm postoperatively. In their study, the number of patients was very small and reliable visual field data were scant. They concluded that cupping reversal in paediatric glaucoma may not predict improved health of the optic nerve head. Chang and Grajewski reported paradoxical RNFL thinning in a 5-year-old female showing optic disc cupping reversal after IOP reduction by trabeculotomy, from 49 mmHg to 18 mmHg^11^. We suggest that the important similarity between these reports and our study is the very high preoperative IOP.

Lee & Kim^8^ reported factors associated with the rate of postoperative average RNFL thinning after trabeculectomy. They calculated the rate of RNFL thinning using SD-OCT data obtained at between 6 months and 2.5 years after surgery; we used postoperative SD-OCT data obtained at 3–6 months. In their study, shorter follow-up duration after surgery and higher preoperative IOP, defined as the average of two measurements made within 2 weeks before surgery and ranging from 12–44 mmHg, were significantly associated with a faster rate of RNFL thinning. As a possible explanation, they suggested that patients with a long-term follow-up may have received more intensive treatment when glaucoma progression was detected, but they did not present preoperative IOP data. We suggest that recovery of peripapillary RNFL swelling, which is caused by a high IOP^31–33^. could represent an additional explanation for these results. This recovery of swelling, reducing RNFL thickness, may seem like rapidly worsening glaucoma. Subsequently, the longer the follow up duration, the slower the rate of RNFL thinning may appear regardless of the presence of glaucomatous changes.

Swelling of the peripapillary RNFL during acute IOP elevation may be caused not only by mechanical damage from total blockage of axoplasmic flow in some axons but also by ischemic damage resulting from compression of prelaminar vessels^31,33^. Regarding the extent of primary insult, less severe damage may result in remission of swelling and prevention of retinal ganglion cell death, whereas more severe damage may result in progressive injury and retinal ganglion cell death^34–38^. The increase in RNFL thickness with highly elevated IOP, and sequential decrease in RNFL thickness following IOP-lowering treatment, has previously been described in acute angle-closure glaucoma (AACG)^39–43^. A longer duration of IOP elevation (>48 h vs. <48 h)^41,42^ and higher IOP measured during an acute episode (>50 mmHg vs. > 21 mmHg)^41,43^ were both associated with greater RNFL thinning after acute angle-closure attack. Changes in RNFL thickness plateaued by 3 months after the IOP had returned to normal values^40^. RNFL thickening due to high IOP may not be limited only to AACG, but also be possible in conditions characterised by a high IOP of sufficient duration, regardless of the type of glaucoma. Our patients had heterogeneous types of glaucoma. In subgroup analyses of POAG patients and those with other types of glaucoma, both groups showed significant RNFL thinning after surgery, as well as a significant positive correlation between their preoperative peak IOP and postoperative thinning of the RNFL.

In this study, preoperative average RNFL thickness was also associated with postoperative RNFL thinning. The thicker the baseline RNFL, the faster the rate of RNFL thinning in glaucoma^44,45^. Based on our results, it is possible that having more retinal nerve fibres remaining that can cause oedema under very high IOP conditions results in a thicker RNFL before surgery. In the present study, the average GCIPL thickness showed no reduction among all patients (p = 0.145). In subgroup analyses of POAG versus glaucoma other than POAG, there was no reduction in the average GCIPL thickness (p = 0.353 and p = 0.138, respectively). In the subgroup with a higher preoperative peak IOP ≥ 37 mmHg, the average GCIPL thickness showed modest but significant thinning (2.4 µm, p = 0.030). Either recovery of the less severe swelling seen in GCIPL, or delayed thinning with respect to retinal ganglion cell death, could explain these changes. Based on the lack of deterioration of the visual field and a smaller change in GCIPL thickness relative to RNFL thickness (2.4 µm vs. 9.7 um), we speculate that the RNFL thinning after surgery is not due entirely to deterioration of glaucoma, instead involving the recovery of swelling. In other words, in patients with very high preoperative IOPs, RNFL thickness measured preoperatively may mask the extent of existing optic neuropathy.

In this study, enrolled patients were required to have a postoperative mean IOP of <22 mmHg to reduce the possibility of additional postoperative glaucomatous damage by uncontrolled IOP. In addition, we compared the average RNFL and average GCIPL thicknesses measured at postoperative 10–12 months with those measured at postoperative 3–6 months (see Supplementary Fig. S1). There was no significant change in average RNFL or GCIPL thickness (p = 0.109 and p = 0.409, respectively). The postoperative mean IOP at 10–12 months was 15.9 ± 4.5 mmHg, which was not significantly different from the postoperative mean IOP at 6–8 months (14.8 ± 3.5 mmHg, paired *t*-test, p = 0.412). In both subgroups, preoperative peak IOPs ≥ 37 mmHg and < 37 mmHg, and RNFL thickness measured at postoperative 10–12 months, did not differ from those measured at postoperative 3–6 months (p = 0.279 and p = 0.229, respectively, see Supplementary Fig. S2). It is therefore unlikely that significant glaucomatous damage occurred after surgery, although some patients underwent secondary surgery, such as cataract surgery, bleb revision, or cyclophotocoagulation during the interim period.

### Study limitations

This study had several limitations. First, because of its retrospective design, there was a time lag of up to 3 months between postoperative SD-OCT and visual field tests. Although the lack of changes in the visual field results after 3 months may reinforce the possibility of “pseudo-progression” of glaucoma rather than “true-glaucomatous progression”; false-negative results are possible given the high variability of the visual field test. Second, because we enrolled patients with heterogeneous types of glaucoma, i.e., glaucoma other than POAG, in subgroup analyses, the results for this group are not applicable to any specific type of glaucoma. However, we assume that our findings are not limited to a specific type of glaucoma. Third, there are other possible causes of optic disc damage during the early postoperative period, such as ocular decompression retinopathy. However, in our 60 patients there were no findings typical of ocular decompression retinopathy, such as retinal haemorrhage at the posterior pole, which occurs in 92% of such patients^46^. As a potential mechanism of ocular decompression retinopathy, sudden relief of the high IOP compressing the lamina cribrosa could induce an anterior shift and expansion of the lamina cribrosa, resulting in decreased axoplasmic flow and thus exacerbation of disc oedema. This oedema could compress the central retinal vein, and cause diffuse retinal haemorrhage similar to that seen in central retinal vein occlusion^47,48^. Fourth, not only the magnitude of the IOP but also the duration of high IOP necessary to induce RNFL thickening are important parameters^30–33^. However, we could not identify the duration of high IOP in our patients, because in most types of glaucoma there are no distinct symptoms associated with IOP elevation, unlike in AACG patients. We considered the highest IOP in the month before surgery to be the preoperative IOP, so even if the IOP dropped during medical treatment it remains possible that the IOP had already been high enough to cause optic disc oedema.

In conclusion, although we did not determine the critical value at which IOP induced oedema of the peripapillary RNFL, in patients with a very high preoperative IOP it is important to consider that RNFL thickness may decrease within a few months after surgery, even if the IOP is well controlled. Clinicians need to be more cautious when deciding whether to augment glaucoma treatment when thinning of RNFL thickness occurs within a few months of surgery, unless the postoperative IOP is high. Postoperatively stable GCIPL thickness and visual field results may be helpful in differentiating between glaucoma progression and improvement of RNFL oedema. In a clinical study of changes in glaucoma parameters before versus after IOP-lowering treatment, individual analyses would be required according to whether the IOP before treatment was very high.

## Methods

This retrospective, observational case series was approved by the Institutional Review Board of Chungnam National University Hospital, which waived the requirement for informed consent from participants. It was conducted in accordance with the requirements of the Declaration of Helsinki. Glaucoma patients who underwent trabeculectomy or Ahmed valve implantation for medically uncontrolled high IOP were consecutively enrolled in our glaucoma clinic from July 1, 2013 to July 31, 2016.

In our clinic, all patients undergoing glaucoma surgery routinely undergo a thorough ophthalmic examination within the 2 weeks before surgery, including measurement of best-corrected visual acuity (BCVA), auto-refractometry, slit-lamp biomicroscopy, Goldmann applanation tonometry, gonioscopy, and dilated fundus examination. Dilated fundus photography, SD-OCT (Cirrus HD OCT; Carl Zeiss Meditec, Dublin, CA, USA), and 24–2 Swedish interactive threshold algorithm standard perimetry (Humphrey Field Analyzer II; Carl Zeiss Meditec) are also performed. In the early postoperative period, patients routinely visit our outpatient department at 1 week, 2 weeks, 1 month, 2 months, 3–4 months, and 6–8 months. At each visit, BCVA is checked, and slit lamp biomicroscopy (including an optic disc examination) and Goldmann applanation tonometry are performed. Dilated fundus photography and SD-OCT are performed at 3–6 months postoperatively and a visual field examination is performed at 6–8 months postoperatively.

Trabeculectomy or Ahmed valve implantation is performed for glaucoma patients who have uncontrolled IOP > 20 mmHg after maximally tolerable medical therapy, and in patients expecting and/or experiencing progression of glaucomatous optic neuropathy. This study included patients who had various types of glaucoma, defined as the presence of glaucomatous optic nerve damage (focal thinning or notching of the neuroretinal rim, and a RNFL defect), but those with accompanying diseases affecting the retina or optic disc other than glaucoma were excluded. Patients with extensive peripapillary atrophy that included the optic disc scan circle, i.e., 3.46 mm in diameter in SD-OCT images, were also excluded. Patients with a BCVA < 10/20 Snellen, a history of previous intraocular surgery other than cataract surgery that was performed more than 6 months previously, or who underwent any type of intraocular surgery during the study period were excluded. Patients with postoperative mean IOP ≥ 22 mmHg, or with a sustained (>1 month) postoperative IOP lower than 6 mmHg regardless of the presence of a hypotony-associated symptom or sign were excluded. If both eyes of a patient were eligible for inclusion, the eye with the higher preoperative peak IOP was selected.

### IOP parameters

Preoperative peak IOP was defined as the highest IOP among all those measured preoperatively within 1 month before surgery, regardless of the number and type of IOP-lowering medications used for treatment. The postoperative mean IOP was calculated by adding together all of the IOPs recorded during the postoperative period commencing 1 month after surgery until the postoperative ophthalmic examination, which included fundus photography, SD-OCT, and visual field examinations (6–8 months), and then dividing by the total number of IOP examinations. The IOP reduction was the difference between the preoperative peak IOP and postoperative mean IOP, and IOP fluctuation was defined as the standard deviation of the postoperative mean IOP.

### Peripapillary RNFL and macular GCIPL thickness parameters

Optic disc scans using the 200 × 200 optic disc cube protocol for peripapillary RNFL thickness measurements and macular scans using the 512 × 128 macular cube protocol for macular GCIPL thickness measurements were performed using a Cirrus HD OCT instrument. All OCT scan results were reviewed by a glaucoma specialist. Preoperative and postoperative images with a signal strength ≥6, and an absence of any artefacts caused by eye motion, blinking, poor centration, or segmentation error, were included in the statistical analyses. The evaluated RNFL thickness parameters included average thickness (360° measurement) and the thickness of four quadrants (temporal, superior, nasal, and inferior quadrant). The average GCIPL thickness and thickness of six sectors (superotemporal, superior, superonasal, inferonasal, inferior, and inferotemporal) in the macular elliptical annulus were included as GCIPL thickness parameters.

### Statistical analyses

Comparisons between the preoperative and postoperative values for clinical parameters including IOP, RNFL thickness, GCIPL thickness, and visual field test results were performed using a paired *t*-test. For each patient, the change in average RNFL thickness was calculated as the preoperative average RNFL thickness minus the postoperative average RNFL thickness. Univariate linear regression analyses were used to investigate clinical factors associated with RNFL thinning. Multivariate linear regression analyses applying stepwise selection (variables with p > 0.1 removed) was performed, including only variables with a p-value < 0.05 in the univariate model. In subgroup analyses, patients were divided into two groups based on their preoperative peak IOP (median, ≥37 mmHg or <37 mmHg), and according to glaucoma type (primary open-angle glaucoma [POAG] and glaucoma other than POAG). Comparisons between these two groups in terms of demographics and changes in clinical parameters following surgery were performed using Student’s *t*-test and Fisher’s exact test. All statistical analyses were performed using SPSS for Windows statistical software (ver. 18.0; SPSS Inc., Chicago, IL, USA). A value of p < 0.05 was considered statistically significant.

## Supplementary information

Supplement 1

Supplement 2
