# Supplementary material for: Relationship between preoperative high intraocular pressure and retinal nerve fibre layer thinning after glaucoma surgery
Source: Sci Rep. 2019 Sep 25;9:13901. doi: 10.1038/s41598-019-50406-7 (PMC6761197; doi:10.1038/s41598-019-50406-7)
Supplement: Supplementary file 1 — Supplement 1 [file 41598_2019_50406_MOESM1_ESM.pdf]

Relationship between preoperative high intraocular pressure and retinal nerve fibre layer thinning after glaucoma surgery

Woo-Jin Kim, Kyoung Nam Kim, Jae Yun Sung, Jung Yeul Kim, Chang-sik Kim

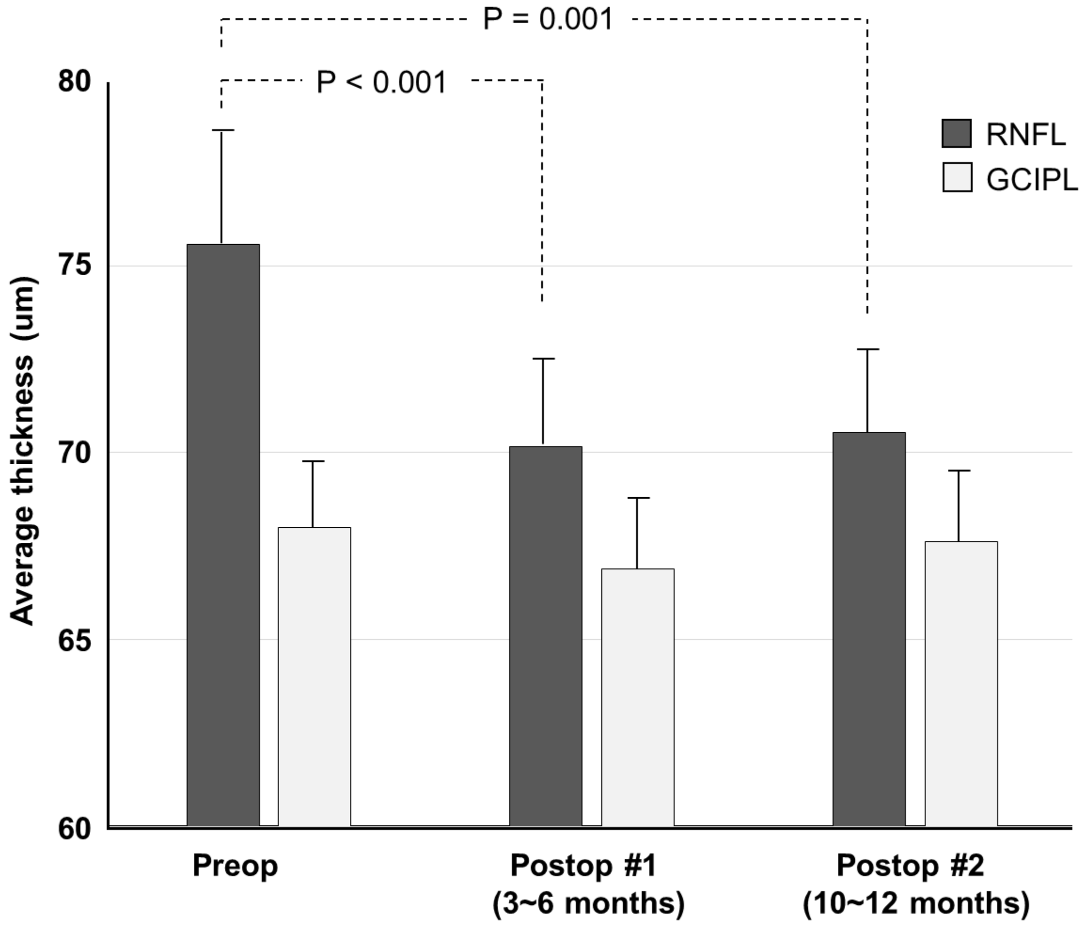

Supplement 1. Averageretinal nerve fiber layer (RNFL) thickness and averageganglion cell-inner plexiform layer (GCIPL) thickness preoperatively(Preop), and at 3~6 monthspostoperatively(Postop #1), and at 10~12 months postoperatively(Postop #2). There were significant differences between Preop RNFL thickness and Postop #1 RNFL thickness ($p < 0.001$),and between Preop RNFL thickness and Postop #2 RNFL thickness ($p=0.001$). However, there was no significant difference between Postop #1RNFL thickness and Postop #2 RNFL thickness ($p=0.109$). The GCIPL thicknessesdid not show any significant change; Preop vs. Postop #1 ($p = 0.145$), Preop vs. Postop #2 ($p = 0.659$), and Postop #1 vs. Postop #2 ($p=0.409$).
