# Supplementary material for: Relationship between preoperative high intraocular pressure and retinal nerve fibre layer thinning after glaucoma surgery
Source: Sci Rep. 2019 Sep 25;9:13901. doi: 10.1038/s41598-019-50406-7 (PMC6761197; doi:10.1038/s41598-019-50406-7)
Supplement: Supplementary file 2 — Supplement 2 [file 41598_2019_50406_MOESM2_ESM.pdf]

Relationship between preoperative high intraocular pressure and retinal nerve fibre layer thinning after glaucoma surgery

Woo-Jin Kim, Kyoung Nam Kim, Jae Yun Sung, Jung Yeul Kim, Chang-sik Kim

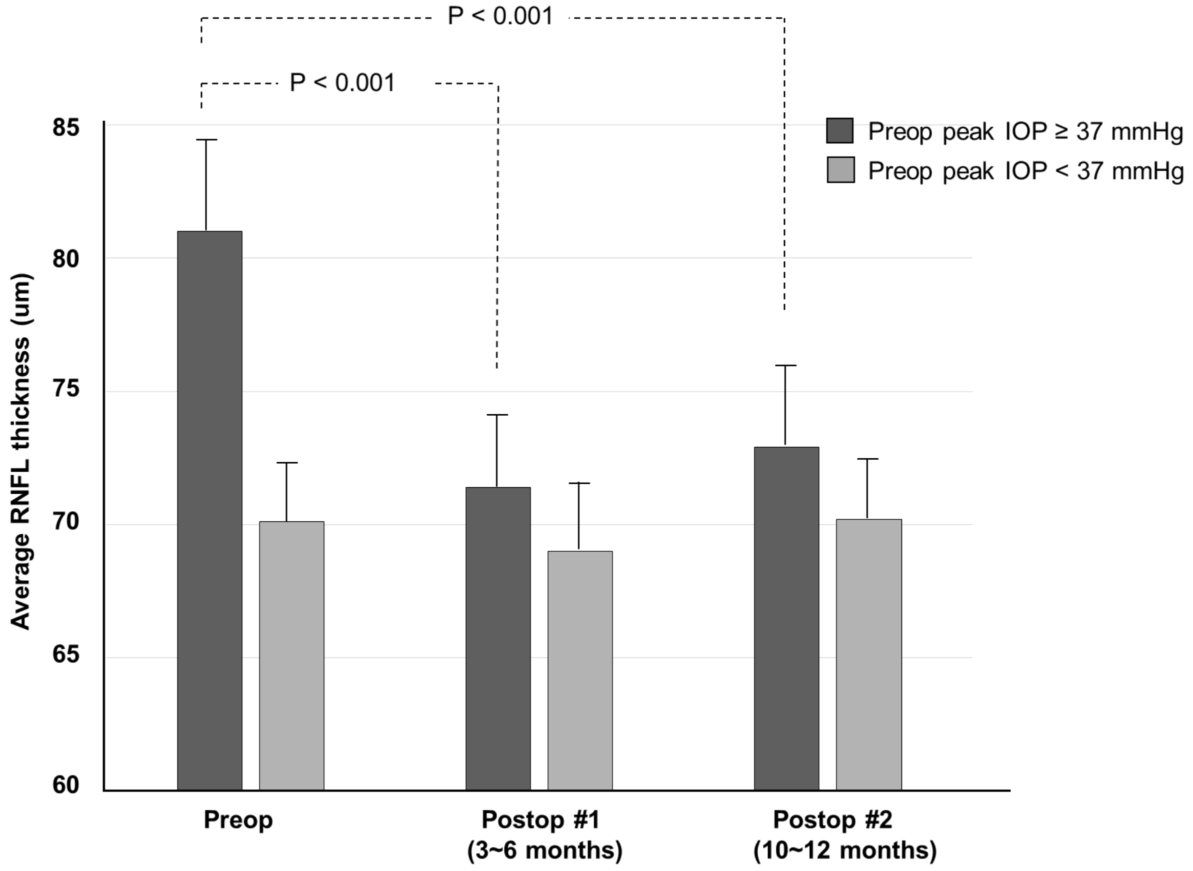

Supplement 2. Average RNFL thickness in patients with preoperative peak intraocular pressure (IOP) ≥37 mmHg (Preop peak IOP ≥ 37 mmHg), and in patients with preoperative peak IOP <37 mmHg (Preop peak IOP < 37 mmHg), preoperatively (Preop) and at 3~6 months (Postop #1) and at 10~12 months postoperatively (Postop #2). In the Preop peak IOP ≥ 37 mmHg group, there were significant differences between Preop and Postop #1 ($p < 0.001$), and between Preop and Postop #2 ($p < 0.001$). However, there was no significant difference between Postop #1 and Postop #2 ($p=0.279$). In the Preop peak IOP < 37 mmHg group, the RNFL thickness did not show any significant change; Preop vs. Postop #1 ($p=0.113$), Preop vs. Postop #2 ($p=0.935$), or Postop #1 vs. Postop #2 ($p = 0.229$).
